# Opium use during pregnancy and risk of preterm delivery: A population-based cohort study

**DOI:** 10.1371/journal.pone.0176588

**Published:** 2017-04-27

**Authors:** Siavash Maghsoudlou, Sven Cnattingius, Scott Montgomery, Mohsen Aarabi, Shahriar Semnani, Anna-Karin Wikström, Shahram Bahmanyar

**Affiliations:** 1 Clinical Epidemiology Unit, Department of Medicine, Karolinska Institute, Solna, Sweden; 2 Clinical Epidemiology and Biostatistics, School of Medical Sciences, Örebro University, Örebro, Sweden; 3 Department of Epidemiology and Public Health, University College London, United Kingdom; 4 Faculty of Medicine, Mazandaran University of Medical Sciences, Sari, Iran; 5 Faculty of Medicine, Golestan University of Medical Sciences, Gorgan, Iran; 6 Department of Women's and Children's Health, Uppsala University, Uppsala, Sweden; 7 Clinical Epidemiology Unit & Centre for Pharmacoepidemiology, Department of Medicine, Karolinska Institute, Solna, Sweden; Harvard School of Public Health, UNITED STATES

## Abstract

**Background:**

Use of narcotic or “recreational” drugs has been associated with adverse pregnancy outcomes such as preterm delivery. However, the associations might be confounded by other factors related to high-risk behaviours. This is the first study to investigate the association between traditional opium use during pregnancy and risk of preterm delivery.

**Method and findings:**

We performed a population-based cohort study in the rural areas of the Golestan province, Iran between 2008 and 2010. We randomly selected 920 women who used (usually smoked) opium during pregnancy and 920 women who did not. Logistic regression models were used to estimate odds ratios (OR) and 95% confidence intervals (CI) for the associations between the opium use during pregnancy and preterm delivery and adjustment was made for potential confounding factors. This study shows compared with non-use of opium and tobacco, use of only opium during pregnancy was associated with an increased risk of preterm delivery (OR = 1.56; 95% CI 1.05–2.32), and the risk was more than two-fold increased among dual users of opium and tobacco (OR = 2.31; 95% CI 1.37–3.90). We observed that opium use only was associated with a doubled risk for preterm caesarean delivery (OR = 2.05; 95% CI 1.10–3.82) but not for preterm vaginal delivery (OR = 1.25; 95% CI 0.75–2.07). Dual use of opium and tobacco was associated with a substantially increased risk of vaginal preterm delivery (OR = 2.58; 95% CI 1.41–4.71).

**Conclusions:**

Opium use during pregnancy among non-tobacco smokers is associated with an increased risk of preterm caesarean delivery, indicating an increased risk of a compromised foetus before or during labour. Women who use both opium and smoked during pregnancy have an increased risk of preterm vaginal delivery, indicating an increased risk of spontaneous preterm delivery.

## Introduction

Preterm birth is one of the most important causes of infant mortality.[1] Globally, 14% of the mortality under five years of age is associated with preterm birth.[2] Moreover, preterm birth is a major determinant of short and long-term morbidity, including learning disabilities,[3] respiratory illnesses,[4] hypertension,[5] and cardiovascular events in adulthood.[6] The rate of preterm birth ranges from 5% of live births in northern Europe to 25% in some low and middle-income countries.[7]

Using opioids, including heroin, has been associated with adverse pregnancy outcomes, such as preterm delivery, low birth weight, small for gestational age, and neonatal complications.[8–10] Heroin use during pregnancy has also been associated with increased risks of preeclampsia, third trimester bleeding, foetal distress, and meconium aspiration.[11] However, previously reported associations of opioids and pregnancy outcomes might be confounded by other factors related to high-risk behaviours and lifestyle, including use of other harmful substances, such as tobacco, alcohol, or cocaine.[12] Therefore, it is difficult to distinguish the effects of opioid use from effects of other risk factors.[13]

In Golestan province, northern Iran, some women use (mainly smoke) opium,[14] also during pregnancy. Opium is obtained from the unripe seed capsules of the poppy plant, Papaver somniferum, which contains a number of alkaloids, including Morphine (10% of opium), Codeine (0.5%), Thebaine (0.2%), Papaverine (1%), and noscapine (6%).[15] In this area, opium-using women are mostly housewives, who do not otherwise have an unhealthy lifestyle, which reduces the risk of residual confounding. [14,16,17]

The primary health care organization in the Golestan province provides modern antenatal and obstetric care to virtually all pregnant women. In the rural areas, most pregnancies are planned, and most women have a pre-pregnancy visit followed by several antenatal visits. Details of maternal drug use, cigarette smoking, alcohol use, and other high-risk behaviours before and during pregnancy are recorded.[17]

The aim of this population-based cohort study was to investigate the association between maternal opium use during pregnancy and risk of preterm delivery in a homogeneous population from rural areas in north-east Iran.

## Methods

### Study setting

The study was performed in Golestan province, in northeast Iran. Golestan has approximately 1,700,000 inhabitants (50% living in rural areas), 500,000 women in reproductive age and 17,000 births annually. Based on the public health care system in Iran, each village has at least one rural health house, which is responsible for providing care and recording of information before and during pregnancy. Health care providers in approximately 600 health houses prospectively record such information in the family health files. Some 95% of mothers deliver their infants in hospitals.[18] Information about delivery (time and date, gestational age, mode of delivery, and complications at delivery) and infant characteristics (sex, weight, height, and head circumference at birth, as well as Apgar scores at one and five minutes) is sent from the hospitals to the health houses for archiving in family health files.

### Study population and design

We performed a population-based cohort study in rural areas of Golestan province between 2008 and 2010. Exposure was defined as self-reported history of using any variety of opiates during the index pregnancy. We lacked information on the form of opium use. Opium (opium poppy and derivatives of opium i.e. tincture, syrup, extract, and opium powder) is the most common drug in Golestan province, and is usually smoked. Very few people drink or eat some of its derivates (such tincture and syrup) and nobody injects opium.[14] All prospectively recorded information in the family health files in rural health houses, including maternal drug use, cigarette smoking, alcohol use, and other high-risk behaviours before and during pregnancy is collected and recorded by the local health officer, who are trained to, among other activities, take care of pregnant women and record the information in family health files and lives in the village as a highly respected person and has personal knowledge about the inhabitants. There is detailed guideline for each of the activity and how to fill in the forms. The unexposed group included women who did not have any positive history of opium use before or during pregnancy.

Exposed and unexposed groups were selected using the stratified randomization sampling method. We determined each region as a block and calculated block sample size based on the population growth rate of the region. All pregnancies during the study period in the region (30,868 pregnancies) were numbered based on date of delivery, and those who were exposed to opium were identified. Exposed and unexposed pregnancies were randomly selected using computer-created random digits. We selected the same number of women exposed and unexposed to opium during pregnancy, who were frequency matched by residential area (village).

Information on maternal and pregnancy characteristics were collected from medical records by midwives working at the health centres. Data were abstracted from pre-pregnancy, pregnancy, and delivery records, and information was computerized by ten specially trained medical students. Each file, including information on the mother, infant, and spouse, received a unique code at the time of data collection, which made it possible to analyse the data anonymously. As a quality control, we collected and computerized 10% of the data a second time. The variables that had more than 5% mismatches were re-collected and the data were re-entered for all study subjects (happened for the Estimated Date of Confinement). If information for each variable for a health centre had more than 5% mismatches, information on all variables were re-collected and re-entered (happened for one health centre).

We selected a total of 920 opium users (“exposed” women) and 920 non-opium users (“unexposed” women) during pregnancy. Thereafter, we excluded 10 exposed and 11 unexposed women due to multiple births, miscarriages, and stillbirths. We also excluded 18 (exposed) mothers who gave up using opium in early pregnancy. We also excluded, two mothers unexposed to opium but exposed to tobacco, and mothers who used alcohol during pregnancy (2 unexposed and 5 exposed mothers), from the analysis due to paucity of the numbers and for increasing internal validity. Finally, our study included 887 opium users and 905 non-opium users with live singleton births.

Information on socioeconomic situation was based on husband’s profession (categorized as unskilled manual worker, skilled manual worker, self-employed, farmer, other occupations, and unemployed). Agricultural production is the main economic sector in Golestan province. Small for gestational age was defined as a birth weight below the 10^th^ percentile for appropriate gestational age, based on a sex-specific global reference for birth weight.[19]

Gestational age was based on the time interval between date of delivery and date of the first day of the last menstrual period. Preterm delivery was defined as a delivery before 37 completed gestational weeks.

This work was entirely funded by internal funding of Karolinska Instituten, grant recipient Associate Professor Shahram Bahmanyar. The study was approved by the Research Ethics Committee of Golestan University of Medical Sciences and the Karolinska Institutet regional ethics committee. As only anonymized previously recorded information was used in this study, informed consent was not needed.

### Statistical analysis

We used univariate and multivariate logistic regression models to estimate odds ratios (ORs) and 95% confidence intervals (CIs) for the associations between opium use during pregnancy and preterm delivery. The multivariate models were adjusted for residential place, maternal age, body mass index, height, parity, husband’s occupation, and infant sex (model A). We lacked specific information on spontaneous and medically indicated preterm delivery. Therefore, risks of vaginal preterm delivery (predominantly including spontaneous preterm deliveries) and caesarean section preterm delivery (including both medically indicated preterm deliveries and emergency caesarean deliveries) were investigated separately. To investigate whether a possible association between opium use and caesarean preterm delivery (which included medically indicated caesarean preterm deliveries) was mediated by maternal hypertensive disorders or intrauterine growth restriction, we additionally adjusted for birth of a small for gestational age infant and any record of hypertension during pregnancy (model B). Additional analyses were performed to investigate the risks of preterm delivery among opium-using mothers who smoked or did not smoke tobacco during pregnancy.

The multiple imputation method was used to impute missing recorded values for maternal age (6 among mothers unexposed to opium and 5 among exposed mothers), infant sex (15 among mothers unexposed to opium and 10 among exposed mothers), husband’s occupation (4 among unexposed and 9 among exposed), and mother’s height (39 among unexposed and 17 among exposed). The MI procedure (SAS software) with five imputations was used for multiple imputation[20]. SAS software version 9.4 was used for all analyses.

## Results

Characteristics of the opium users and non-opium users are presented in Table 1. Opium users were slightly older and more often parous compared with non-opium users. There were no noteworthy differences between the two groups regarding maternal body mass index or husband’s occupation.

**Table 1:** Characteristics of study subjects.

	Unexposed^1^	Opium user			
		All	Opium user only	Tobacco and Opium user	
	N (%)	N (%)	N (%)	N (%)	ρ-value^2^
	905 (100)	887 (100)	677 (100)	210 (100)	
**Maternal age (Years)**					< .0001
≤ 19	134 (14.8)	49 (5.5)	40 (5.9)	9 (4.3)	
20–24	250 (27.6)	161 (18.2)	133 (19.6)	28 (13.3)	
25–29	260 (28.7)	278 (31.3)	210 (31.0)	68 (32.4)	
30–34	187 (20.7)	251 (28.3)	181 (26.7)	70 (33.3)	
≥ 35	68 (7.5)	143 (16.1)	108 (16.0)	35 (16.7)	
**Maternal height (cm)**					0.9272
≤ 149	68 (7.5)	66 (7.4)	47 (6.9)	19 (9.0)	
150–155	198 (21.9)	193 (21.8)	152 (22.5)	41 (19.5)	
156–160	380 (42.0)	399 (45.0)	295 (43.6)	104 (49.5)	
161–164	120 (13.3)	109 (12.3)	84 (12.4)	25 (11.9)	
≥ 165	100 (11.0)	103 (11.6)	86 (12.7)	17 (8.1)	
**Maternal body mass index**					0.4830
< 18.5	63 (7.0)	52 (5.9)	34 (5.0)	18 (8.6)	
18.5 to < 25	439 (48.5)	474 (53.4)	356 (52.6)	118 (56.2)	
25 to < 30	245 (27.1)	231 (26.0)	184 (27.2)	47 (22.4)	
30 to < 35	91 (10.1)	91 (10.3)	72 (10.6)	19 (9.0)	
≥ 35	28 (3.1)	21 (2.4)	18 (2.7)	3 (1.4)	
**Parity**					< .0001
Nulliparous	372 (41.1)	199 (22.4)	155 (22.9)	44 (21.0)	
Multiparous	533 (58.9)	688 (77.6)	522 (77.1)	166 (79.0)	
**Delivery method**					0.2541
Vaginal delivery	559 (61.8)	572 (64.5)	428 (63.2)	144 (68.6)	
Caesarean section	320 (35.4)	292 (32.9)	234 (34.6)	58 (27.6)	
**Husband’s occupation**					0.0023
Unemployed	25 (2.8)	56 (6.3)	37 (5.5)	19 (9.0)	
Non skill worker	400 (44.2)	419 (47.2)	312 (46.1)	107 (51.0)	
Skill worker	91 (10.1)	70 (7.9)	58 (8.6)	12 (5.7)	
Self-employed	111 (12.3)	101 (11.4)	89 (13.1)	12 (5.7)	
Farmer	179 (19.8)	163 (18.4)	123 (18.2)	40 (19.0)	
Other	95 (10.5)	69 (7.8)	52 (7.7)	17 (78.1)	
**Hypertension during pregnancy**					0.8906
Yes	28 (3.1)	26 (2.9)	19 (2.8)	7 (3.3)	
No	877 (96.9)	861 (97.1)	658 (97.2)	203 (96.7)	
**Gestational age weeks (weeks)**					< .0001
Mean (SD)	39.3 (1.6)	39.1 (1.9)	39.0 (1.9)	38.7 (2.1)	
Median (Range)	39 (29–43)	39 (28–42)	39 (29–42)	39 (28–42)	
**Birth of an infant small for gestational age**					< .0001
Yes	105 (11.6)	164 (18.5)	125 (18.5)	39 (18.6)	
No	785 (86.6)	713 (80.2)	543 (80.2)	170 (81.0)	

^1^Unexposed includes mothers who are non-users of both opium and tobacco.

^2^ρ-value of unexposed group comparing with all exposed.

The rates of preterm delivery among mothers who used and did not use opium during pregnancy were 10.0% and 6.1%, respectively. Compared with unexposed mothers (unexposed to both opium and tobacco smoking), mothers exposed to opium use during pregnancy had a 74% higher risk of preterm delivery (Table 2, model A). Compared with unexposed mothers, non-smoking mothers who used opium had a 56% increased risk for preterm delivery and those who used both opium and tobacco during pregnancy had a more than two-fold increased risk. When we additionally adjusted for hypertension during pregnancy and small for gestational age, results were essentially unchanged (Table 2, model B).

**Table 2:** Odds ratios (OR) and 95% confidence intervals (CI) for association between opium and tobacco use during pregnancy and risk of preterm delivery.

	Pregnancies N	Events		Crude OR (95% CI)	Model A^1^ OR (95% CI)	Model B^2^ OR (95% CI)
		N	(%)			
**Opium**						
Unexposed	905	55	(6.1)	Reference		
Exposed	887	89	(10.0)	1.73 (1.22–2.45)	1.74 (1.21–2.45)	1.69 (1.16–2.44)
**Tobacco and opium**						
Unexposed	905	55	(6.1)	Reference		
Exposed to opium only	677	61	(9.0)	1.53 (1.05–2.23)	1.56 (1.05–2.32)	1.51 (1.01–2.24)
Exposed to tobacco and opium	210	28	(13.3)	2.38 (1.47–3.85)	2.40 (1.43–4.04)	2.37 (1.40–3.99)

^1^Adjusted for: maternal age, height, BMI, parity, husband’s occupation, infant sex, delivery method, and residential place.

^2^Adjusted for all covariates which are included in Model A plus hypertension during the pregnancy and small for gestational age.

Next, preterm delivery was divided into vaginal and caesarean preterm deliveries. Compared with unexposed mothers, the risk of vaginal preterm delivery was not significantly increased among opium using mothers (Table 3). Compared with unexposed mothers, those only using opium during pregnancy were not at increased risk of vaginal preterm delivery, but dual use of opium and tobacco was associated with a 2.5-fold increase in risk (Table 3, model A). Also adjusting for hypertension during pregnancy and small for gestational age did not change the association between opium use and vaginal preterm delivery (Table 3, model B).

**Table 3:** Odds ratios (OR) and 95% confidence intervals (CI) for association between opium and tobacco use during pregnancy and risk of vaginal preterm delivery^1^.

	Pregnancies N	Events		Crude OR (95% CI)	Model A^2^ OR (95% CI)	Model B^3^ OR (95% CI)
		N	(%)			
**Opium**						
Unexposed	887	37	(4.2)	Reference		
Exposed	853	55	(6.4)	1.59 (1.03–2.43)	1.45 (0.91–2.31)	1.49 (0.93–2.37)
**Tobacco and opium**						
Unexposed	887	37	(4.2)	Reference		
Exposed to opium only	649	33	(5.1)	1.23 (0.76–1.99)	1.25 (0.75–2.07)	1.28 (0.77–2.13)
Exposed to tobacco and opium	204	22	(10.8)	2.78 (1.60–4.82)	2.58 (1.41–4.71)	2.69 (1.47–4.97)

^1^ Those with caesarean section preterm delivery are excluded

^2^Adjusted for: maternal age, height, BMI, parity, husband’s occupation, infant sex, and residential place.

^3^Adjusted for all covariates which are included in Model A plus hypertension during the pregnancy and small for gestational age.

Overall, opium use during pregnancy was associated with a doubled risk of caesarean section preterm delivery (Table 4, model A). Compared with non-use of opium and tobacco, use of only opium was associated with a doubled risk of caesarean section preterm delivery. The corresponding risk related to dual use of opium and tobacco was not statistically significantly increased, but the analysis was limited by a small number of events (Table 4). When we also adjusted for hypertension during pregnancy and small for gestational age, the association between opium use and caesarean section preterm delivery was attenuated: the point estimates moved toward the null and did not reach statistical significance.

**Table 4:** Odds ratios (OR) and 95% confidence intervals (CI) for association between opium and tobacco use during pregnancy and risk of caesarean section preterm delivery^1^.

	Pregnancies N	Events		Crude OR (95% CI)	Model A^2^ OR (95% CI)	Model B^3^ OR (95% CI)
		N	(%)			
**Opium**						
Unexposed	868	18	(2.1)	Reference		
Exposed	832	34	(4.1)	2.02 (1.13–3.60)	1.99 (1.09–3.64)	1.69 (0.92–3.13)
**Tobacco and opium**						
Unexposed	868	18	(2.1)	Reference		
Exposed to opium only	644	28	(4.3)	2.15 (1.18–3.92)	2.05 (1.10–3.82)	1.75 (0.93–3.29)
Exposed to tobacco and opium	188	6	(3.2)	1.56 (0.61–3.98)	1.75 (0.65–4.70)	1.46 (0.53–4.01)

^1^Those with preterm vaginal delivery are excluded.

^2^Adjusted for: maternal age, height, BMI, parity, husband’s occupation, infant sex, and residential place.

^3^Adjusted all covariates which are included in Model A plus hypertension during the pregnancy and small for gestational age.

## Discussion

To the best of our knowledge, this is the first study of traditional opium use during pregnancy and risk of preterm delivery. We found that opium use during pregnancy was associated with an increased risk of preterm delivery. The risk was apparent for opium users during pregnancy who were non-tobacco smokers, and was even higher for women exposed to both opium and tobacco during pregnancy.

For opium users who did not use tobacco, the risk was apparent for caesarean section preterm delivery. This group included both elective (pre-labour medically indicated) preterm caesarean deliveries due to concern about maternal or foetal wellbeing, and emergency caesarean section deliveries, probably predominantly due to foetal asphyxia and lack of labour progress.[21] Maternal hypertensive diseases [22] and foetal asphyxia, causing growth restriction, could be on the causal pathway from maternal opium use to both elective and emergency caesarean deliveries. We observed that the association between opium use and caesarean preterm delivery was attenuated after controlling for maternal hypertensive disorders and small for gestational age. Possibly, opium use during pregnancy may increase the risk of caesarean preterm delivery by inducing foetal stress before or during labour.[23]

Dual use of opium and tobacco during pregnancy was associated with an increased risk of vaginal preterm delivery. As labour is rarely induced in preterm pregnancies, our group of vaginal preterm deliveries most likely primarily included preterm deliveries with a spontaneous onset of labour. Smoking is associated with increased risks of premature rupture of membranes,[24] intrauterine infection, prostaglandin activity in foetal membranes, and an increased systemic inflammatory response, which can lead to spontaneous preterm labour and in consequence preterm delivery.[25] Although tobacco smoking is associated with increased risks of both medically indicated and spontaneous preterm delivery, the association is stronger for spontaneous preterm delivery.[26] The observed more than two-fold increased risk of vaginal preterm delivery in dual users of opium and tobacco is substantially higher than the risk of spontaneous preterm delivery reported for smokers.[26] Thus, residual confounding by tobacco smoking is unlikely to explain our findings. We speculate that the high risk of vaginal preterm delivery among mothers who were exposed to both opium and tobacco during pregnancy could be due to additive or interactive effects of the two risk factors.[8,10]

Different explanations for the associations between use of opiates and risks of adverse pregnancy outcomes have been proposed. The first theory is that maternal narcotic exposure can induce fluctuating cycles of intoxication and withdrawal in the foetus, and that the foetal oxygen needs may not be met during withdrawal. This recurrent intrauterine hypoxic stress can lead to preterm delivery, intrauterine growth restriction, and meconium-stained amniotic fluid.[27] The second explanation is the epiphenomena of drug use, including multiple drug abuse, poor prenatal care, dietary restriction, and disadvantaged socioeconomic circumstances.[28]

Our findings are consistent with results of studies including drug abusers, reporting positive associations between narcotic use, such as heroin, and risk of preterm delivery.[8,9] Pinto et al. showed that mothers using methadone, heroin, or other addictive drugs had an increased risk of preterm delivery but the sample size was not big enough to investigate each drug separately.[10] A British study showed that opiates (heroin or methadone) use during pregnancy was associated with an increased risk of preterm delivery.[8] Although methadone is less harmful compared with other opioids and may confer many advantages for women with an opioid use disorder and their infant, methadone use during pregnancy has been associated with a three-fold increased risk of preterm delivery and an even higher risk was found among mothers who used both methadone and other illicit drugs.[29] However, as most drug abusers have a chaotic lifestyle, it is difficult to estimate the association between specific drug use and adverse pregnancy outcomes in earlier studies.[9]

Opium has traditionally been used in Middle-eastern and south Asian countries such as Iran for pleasure and as a sedative.[30] Compared with other drug abusers, opium users have less psychiatric comorbidity, unemployment, homelessness, and criminal behaviour.[31] It has been shown that the socioeconomic profile of opium users in Iran is similar to that of the general population.[14] The current study was conducted in rural areas of Golestan province, where most mothers are housewives, pregnancies are planned, and approximately 97% of pregnant women have access to the state primary health service free of cost.[18] Adjustment for husband’s occupation and residential place did not affect the observed associations in our data. However, we cannot rule out residual confounding.

### Strengths and limitations

The strengths of this study include the population-based cohort design using prospectively collected information from antenatal visits. As we used prospectively collect information from records of pre-pregnancy, antenatal visits and delivery, and those who abstracted the forms were blinded to the study hypothesis, there is no concern regarding possible observer bias or bias due to factors such as non-blinded exposure or outcome determination. Most previous studies are derived from investigations of mothers exposed to heroin or methadone in high-income countries, while information from traditional opium use in low or middle-income countries is lacking.[13] A general issue in studies in which the effect of illicit drugs are investigated is confounding by factors related to lifestyle, such as multiple drug abuse, alcohol consumption, tobacco smoking, and even blood-borne infectious diseases.[10,32] This study provides an exceptional possibility to investigate the risk of preterm delivery among opium exposed mothers who otherwise had a normal life and an acceptable level of care during pregnancy. To increase internal validity, we limited our study to non-alcohol users, used non-users of opium and tobacco as reference group, and estimated risks among women only using opium and women with dual use of opium and tobacco.

This study has some potential limitations, including lack of information on pregnancies that ended before 28 weeks of gestation. In this study, maternal drug use was based on mothers’ disclosure to their care providers, and unwillingness to admit drug may have pushed our estimates toward to the null. However, a validation study of self-reported opium use with a urine opium detection laboratory test from northern Iran reported reasonably high sensitivity (91%) and specificity (89%).[16] We did not have any information about the mothers’ educational level, nor did we did have information on some potentially important factors that could influence pregnancy outcome, such as mothers’ unhealthy or associated lifestyle factors, including domestic violence, chronic pain due to disease or malnutrition. Estimation of gestational age was based on the first day of last maternal menstrual period, which can be subject to error.[33] Another limitation is that we did not have information on possible exposure to passive opium or tobacco smoking. We also restricted our study to pregnancies in rural area of the Golestan province and there may be concern regarding generalizability of the results. Lastly, a lack of specific information on mothers’ socioeconomic circumstances was also an important potential limitation of this project. Controlling for husband’s occupation and place of residence as a measure of socioeconomic circumstances for the families may not have been sufficiently discriminatory, and we can therefore not rule out effects of residual confounding.

## Conclusion

Opium use during pregnancy is associated with an increased risk of preterm delivery. Opium use alone may be associated with increased risk of medically indicated preterm delivery, while dual use of opium and tobacco during pregnancy may increase the risk of spontaneous preterm delivery.

## References

[1] Kramer MS, Demissie K, Yang H, Platt RW, Sauve R, Liston R. The contribution of mild and moderate preterm birth to infant mortality. Fetal and Infant Health Study Group of the Canadian Perinatal Surveillance System. JAMA: the journal of the American Medical Association. 2000;284(7):843–9. 10938173 10.1001/jama.284.7.843

[2] Liu L, Johnson HL, Cousens S, Perin J, Scott S, Lawn JE, et al Global, regional, and national causes of child mortality: an updated systematic analysis for 2010 with time trends since 2000. The Lancet. 2012;379(9832):2151–61. 10.1016/S0140-6736(12)60560-1 22579125

[3] Reading R. Educational and behavioural problems in babies of 32–35 weeks gestation. Child: Care, Health and Development. 2002;28(1):121–2.

[4] Baraldi E, Filippone M. Chronic lung disease after premature birth. The New England journal of medicine. 2007;357(19):1946–55. 10.1056/NEJMra067279 17989387

[5] de Jong F, Monuteaux MC, van Elburg RM, Gillman MW, Belfort MB. Systematic review and meta-analysis of preterm birth and later systolic blood pressure. Hypertension. 2012;59(2):226–34. 10.1161/HYPERTENSIONAHA.111.181784 22158643 PMC3266458

[6] Kaijser M, Bonamy AK, Akre O, Cnattingius S, Granath F, Norman M, et al Perinatal risk factors for ischemic heart disease: disentangling the roles of birth weight and preterm birth. Circulation. 2008;117(3):405–10. 10.1161/CIRCULATIONAHA.107.710715 18172034

[7] Goldenberg RL, Culhane JF, Iams JD, Romero R. Epidemiology and causes of preterm birth. Lancet. 2008;371(9606):75–84. Epub 2008/01/08. 10.1016/S0140-6736(08)60074-4 18177778 PMC7134569

[8] Fajemirokun-Odudeyi O, Sinha C, Tutty S, Pairaudeau P, Armstrong D, Phillips T, et al Pregnancy outcome in women who use opiates. European journal of obstetrics, gynecology, and reproductive biology. 2006;126(2):170–5. 10.1016/j.ejogrb.2005.08.010 16202501

[9] Boer K, Smit BJ, Huis AM, Hogerzei HV. Substance use in pregnancy: do we care? Acta paediatrica. 1994;83(s404):65–71. 10.1111/j.1651-2227.1994.tb13386.x 7841636

[10] Pinto SM, Dodd S, Walkinshaw SA, Siney C, Kakkar P, Mousa HA. Substance abuse during pregnancy: effect on pregnancy outcomes. European journal of obstetrics, gynecology, and reproductive biology. 2010;150(2):137–41. 10.1016/j.ejogrb.2010.02.026 20227162

[11] Ludlow JP, Evans SF, Hulse G. Obstetric and perinatal outcomes in pregnancies associated with illicit substance abuse. The Australian & New Zealand journal of obstetrics & gynaecology. 2004;44(4):302–6. 15282000 10.1111/j.1479-828X.2004.00221.x

[12] Bandstra ES, Morrow CE, Mansoor E, Accornero VH. Prenatal drug exposure: infant and toddler outcomes. Journal of addictive diseases. 2010;29(2):245–58. 10.1080/10550881003684871 20407980

[13] Keegan J, Parva M, Finnegan M, Gerson A, Belden M. Addiction in pregnancy. Journal of addictive diseases. 2010;29(2):175–91. 10.1080/10550881003684723 20407975

[14] Khademi H, Malekzadeh R, Pourshams A, Jafari E, Salahi R, Semnani S, et al Opium use and mortality in Golestan Cohort Study: prospective cohort study of 50,000 adults in Iran. BMJ. 2012;344:e2502 10.1136/bmj.e2502 22511302 PMC3328545

[15] Brunton LL, Chabner B, Knollmann BC. Goodman & Gilman's the pharmacological basis of therapeutics: McGraw-Hill Medical New York; 2011.

[16] Abnet CC, Saadatian-Elahi M, Pourshams A, Boffetta P, Feizzadeh A, Brennan P, et al Reliability and validity of opiate use self-report in a population at high risk for esophageal cancer in Golestan, Iran. Cancer Epidemiology Biomarkers & Prevention. 2004;13(6):1068–70. 15184266

[17] Maghsoudlou S, Cnattingius S, Aarabi M, Montgomery SM, Semnani S, Stephansson O, et al Consanguineous marriage, prepregnancy maternal characteristics and stillbirth risk: a population-based case-control study. Acta obstetricia et gynecologica Scandinavica. 2015;94(10):1095–101. 10.1111/aogs.12699 26085011

[18] Rashidian A KA, Khabiri R, Khodayari-Moez E, Elahi E, Arab M and Radaie Z. Islamic Republic of Iran's Multiple Indicator Demograpphic and Healh Survey (IrMIDHS). 2010 2010. Report No.

[19] Mikolajczyk RT, Zhang J, Betran AP, Souza JP, Mori R, Gulmezoglu AM, et al A global reference for fetal-weight and birthweight percentiles. Lancet. 2011;377(9780):1855–61. 10.1016/S0140-6736(11)60364-4 21621717

[20] Rubin DB, Schenker N. Multiple Imputation in Health-Care Databases—an Overview and Some Applications. Stat Med. 1991;10(4):585–98. 2057657 10.1002/sim.4780100410

[21] Florica M, Stephansson O, Nordstrom L. Indications associated with increased cesarean section rates in a Swedish hospital. International journal of gynaecology and obstetrics: the official organ of the International Federation of Gynaecology and Obstetrics. 2006;92(2):181–5. 10.1016/j.ijgo.2005.10.016 16364324

[22] Cnattingius S, Villamor E, Johansson S, Edstedt Bonamy AK, Persson M, Wikstrom AK, et al Maternal obesity and risk of preterm delivery. JAMA: the journal of the American Medical Association. 2013;309(22):2362–70. Epub 2013/06/13. 10.1001/jama.2013.6295 23757084

[23] Kyrklund-Blomberg NB, Cnattingius S. Preterm birth and maternal smoking: risks related to gestational age and onset of delivery. American journal of obstetrics and gynecology. 1998;179(4):1051–5. 10.1016/S0002-9378(98)70214-5. 9790397

[24] Lee T, Silver H. Etiology and epidemiology of preterm premature rupture of the membranes. Clin Perinatol. 2001;28(4):721–34. 10.1016/S0095-5108(03)00073-3. 11817185

[25] Surkan PJ, Stephansson O, Dickman PW, Cnattingius S. Previous preterm and small-for-gestational-age births and the subsequent risk of stillbirth. The New England journal of medicine. 2004;350(8):777–85. Epub 2004/02/20. 10.1056/NEJMoa031587 14973215

[26] Cnattingius S. The epidemiology of smoking during pregnancy: smoking prevalence, maternal characteristics, and pregnancy outcomes. Nicotine & tobacco research: official journal of the Society for Research on Nicotine and Tobacco. 2004;6 Suppl 2(Suppl 2):S125–40. 15203816 10.1080/14622200410001669187

[27] Huestis MA, Choo RE. Drug abuse's smallest victims: in utero drug exposure. Forensic Sci Int. 2002;128(1–2):20–30. 10.1016/S0379-0738(02)00160-3. 12208017

[28] Vucinovic M, Roje D, Vucinovic Z, Capkun V, Bucat M, Banovic I. Maternal and neonatal effects of substance abuse during pregnancy: our ten-year experience. Yonsei Med J. 2008;49(5):705–13. 10.3349/ymj.2008.49.5.705 18972589 PMC2615365

[29] Almario CV, Seligman NS, Dysart KC, Berghella V, Baxter JK. Risk factors for preterm birth among opiate-addicted gravid women in a methadone treatment program. American journal of obstetrics and gynecology. 2009;201(3):326 e1–6. Epub 2009/07/28. 19631928 10.1016/j.ajog.2009.05.052

[30] Raisdana F, Nakhjavani AG. The drug market in Iran. Ann Am Acad Polit Ss. 2002;582(1):149–66.

[31] Ghaffari Nejad A, Ziaadini H, Banazadeh N. Comparative Evaluation of Psychiatric Disorders in Opium and Heroin Dependent PatientsThis article has been published in the Journal of Rafsanjan University of Medical Sciences in Persian language. Addiction & Health. 2009;1(1):20–3. 24494078 PMC3905498

[32] Holbrook BD, Rayburn WF. Teratogenic risks from exposure to illicit drugs. Obstet Gynecol Clin North Am. 2014;41(2):229–39. 10.1016/j.ogc.2014.02.008 24845487

[33] Kramer MS, McLean FH, Boyd ME, Usher RH. The validity of gestational age estimation by menstrual dating in term, preterm, and postterm gestations. JAMA: the journal of the American Medical Association. 1988;260(22):3306–8. 3054193

